# A Novel Anatomic Reconstruction for Posterior Tibialis Tendon in Treatment of Flexible Adult‐Acquired Flatfoot Deformity

**DOI:** 10.1111/os.13329

**Published:** 2022-06-10

**Authors:** Yong Hu, Yifan Wang, Zheng Huang, Zhengxun Li, Wenpeng Xu, Dongsheng Zhou, Ning Zhang

**Affiliations:** ^1^ Department of Foot and Ankle Surgery The Second Hospital, Cheeloo College of Medicine, Shandong University Ji'nan P. R. China; ^2^ Department of Orthopaedic Shandong Provincial Hospital, Cheeloo College of Medicine, Shandong University Jinan P. R. China

**Keywords:** Attachment reconstruction, Flatfoot, Posterior tibial tendon, Tendon dysfunction

## Abstract

**Objective:**

To present a novel approach for the anatomic reconstruction of the posterior tibialis tendon (PTT) in restoring plantar insertions and evaluate its efficiency in treating flexible adult‐acquired flatfoot deformity (AAFD) caused by PTT dysfunction.

**Methods:**

For AAFD treatment, a novel PTT reconstruction method was presented. The current study involved 16 patients, including three men, and 13 women, from August 2017 to July 2019. The mean age was 43.2 ± 15.1 years (21–64 years). The innovative PTT repair method was used on all patients. The treatment involved performing a traditional Flexor Digitorum Longus (FDL) transfer in the navicular tuberosity and suturing the plantar insertions to FDL as tension was applied to tighten the plantar structures of the foot. The results were retrospectively analyzed. The clinical outcome was assessed using the pain visual analogue scale (VAS), the satisfaction VAS, and the American Orthopedic Foot and Ankle Society ankle–hindfoot scale (AOFAS‐AH). Isokinetic testing was performed using a dynamometer at 60°/s and 120°/s for inversion/eversion and plantarflexion/dorsiflexion, respectively, to determine the mean peak torque. Radiographic measurements were employed to assess the outcomes.

**Results:**

Bone surgeries combined with the modified anatomic PTT reconstruction were performed on patients with medializing calcaneal osteotomy in 12 (75%) patients and subtalar joint fusion in four (25%) patients. The branch linking to the plantar insertions was detected in every case, with an average width of 3.5 ± 0.8 mm (3.1–4.3 mm). All patients were followed up for the mean of 16.8 ± 1.8 months (range, 15–20 months). The average postoperative functional scores, including pain VAS, satisfaction VAS, total AOFAS‐AH, and all AOFAS‐AH sub‐scales, steadily improved during the follow‐up. In the last follow‐up, isokinetic testing revealed no loss of plantarflexion strength (*p* = 0.350 and 0.098) and significant improvement in the inversion strength (*p* = 0.007 and 0.008) in the operated ankles at 60°/s and 120°/s. Radiographic outcomes, particularly the talar head uncovering, improved significantly after more than a year (*p* < 0.001 for all).

**Conclusions:**

The novel technique for PTT reconstruction in restoring the plantar insertions serves as an effective procedure in treating AAFD caused by PTT dysfunction in terms of delivering a consistent improvement in ankle inversion strength, medial longitudinal arch restoring, and satisfactory clinical outcomes.

## Graphical abstract

A novel anatomic reconstruction of posterior tibialis tendon restores the plantar insertions (Central branch), which are essential for medial longitudinal arch maintaining.

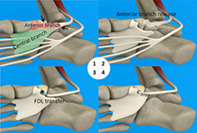

## Introduction

The foot's arch is an essential feature that acts as a shock absorber for weight during walking and standing. The medial longitudinal arch is supported against gravity by both passive and active supports.^1^, ^2^, ^3^, ^4^ Among the active supports, the posterior tibialis is crucial in stabilizing the medial longitudinal arch of the foot, thus supporting the transverse tarsal joint the transverse tarsal joint, the medial longitudinal arch and preventing it from collapsing.^4^, ^5^

Adult‐acquired flatfoot deformity (AAFD) is a condition characterized by progressive hindfoot valgus and the forefoot abduction and varus. The most prevalent cause of this deformity, according to the literature, is posterior tibialis tendon (PTT) dysfunction.^6^, ^7^, ^8^ Initially, the foot is usually arched; however, the spring ligament eventually fails due to prolonged PTT dysfunction and walking stress, and the arch gradually collapses. Persistent hindfoot valgus and forefoot abduction ultimately result in forefoot varus, characterized by the first ray or global forefoot varus at the transverse tarsal joint.

The management of AAFD is determined by the presence or absence of symptoms and the duration.^9^ Numerous techniques have been recommended for many years to treat symptomatic flexible flatfoot. The surgical procedures discussed for the treatment of flatfoot can be divided into two categories: arthrodesis and non‐arthrodesis procedures. Non‐arthrodesis procedures, including soft tissue procedures and bone surgeries, have become the backbone of surgical intervention. The surgical correction should be to the patient's deformity, activity limitations, and adjacent joint arthritis. PTT reconstruction is inextricably linked to adult flexible flatfoot correction surgery, with substantial data supporting the transfer of flexor digitorum longus tendon (FDL) for PTT replacement.^10^, ^11^ The navicular tuberosity is commonly chosen over others because it needs less FDL tendon length and inserting the tendon in this position is supposed to provide more powerful hindfoot inversion and forefoot adduction than the medial cuneiform. Hui and colleagues reported a 46% decreased moment arm of FDL when transferred to the navicular and a 56% decrease when transferred to the medial cuneiform.^12^

PTT exhibits high morphological variability, and its insertion is not clearly defined.^13^, ^14^ The PTT has three primary insertions: an anterior insertion onto the navicular tuberosity, one insertion that extends across the plantar aspects of the remaining tarsal bones and the bases of the middle three metatarsals, and one on the sustentaculum tali. The anatomical findings confirm that the central band is a vital contribution to maintaining the foot's longitudinal arch.^13^ The existing techniques only reconstruct the anterior branch, the subsequent branches are ignored.^10^, ^11^ Herein, the aims of this study were: (i) to introduce a modified PTT anatomic reconstruction in restoring plantar insertions; and (ii) to evaluate its efficiency in treating AAFD using functional scores, isokinetic strength test, and radiographic assessment.

## Methods

### Inclusion and exclusion criteria

The patient inclusion criteria were as follows: (i) age > 14 years and presence of tarsal and calcaneal growth plate closure; (ii) the patient experienced pain along with PTT and flexible deformity with hindfoot valgus, forefoot pronation, and abduction; and (iii) experienced failed conservative treatments with anti‐inflammatory medications, rest, shoe modifications, and foot orthoses over the past 6 months.

The patient exclusion criteria were as follows: (i) patients with neuropathic arthropathy and seropositive arthritis; (ii) patients with comorbidities including venous insufficiency and diabetes; (iii) patients with rigid deformity; or (iv) patients who had undergone surgery at the hindfoot.

### Standard protocol approvals and patient consents

The Hospital Ethics Committee authorized all human studies (No. KYLL‐2019(KJ)‐0155) and performed following the ethical standards. Written informed consent was obtained from all patients.

### General information

**Fig. 1:**
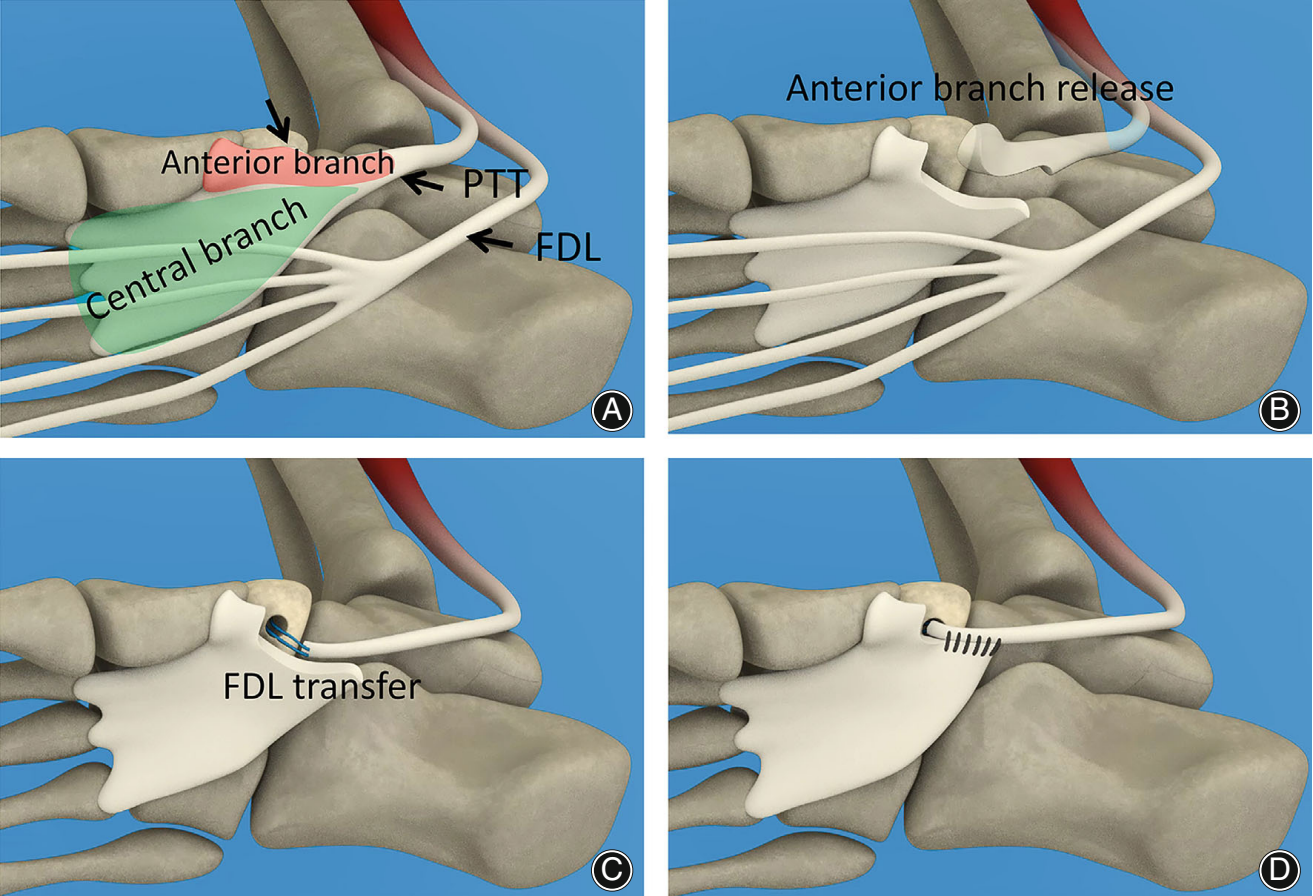
The procedures of the modified PTT reconstruction (A–D). The PTT included insertions involving the anterior and central branches (A). The anterior branch of PTT was released from the attachment (B), followed by the FDL transfer (C). Finally, the central band was sutured to FDL as tension was applied to tighten the plantar structures of the foot (D)

**Fig. 2:**
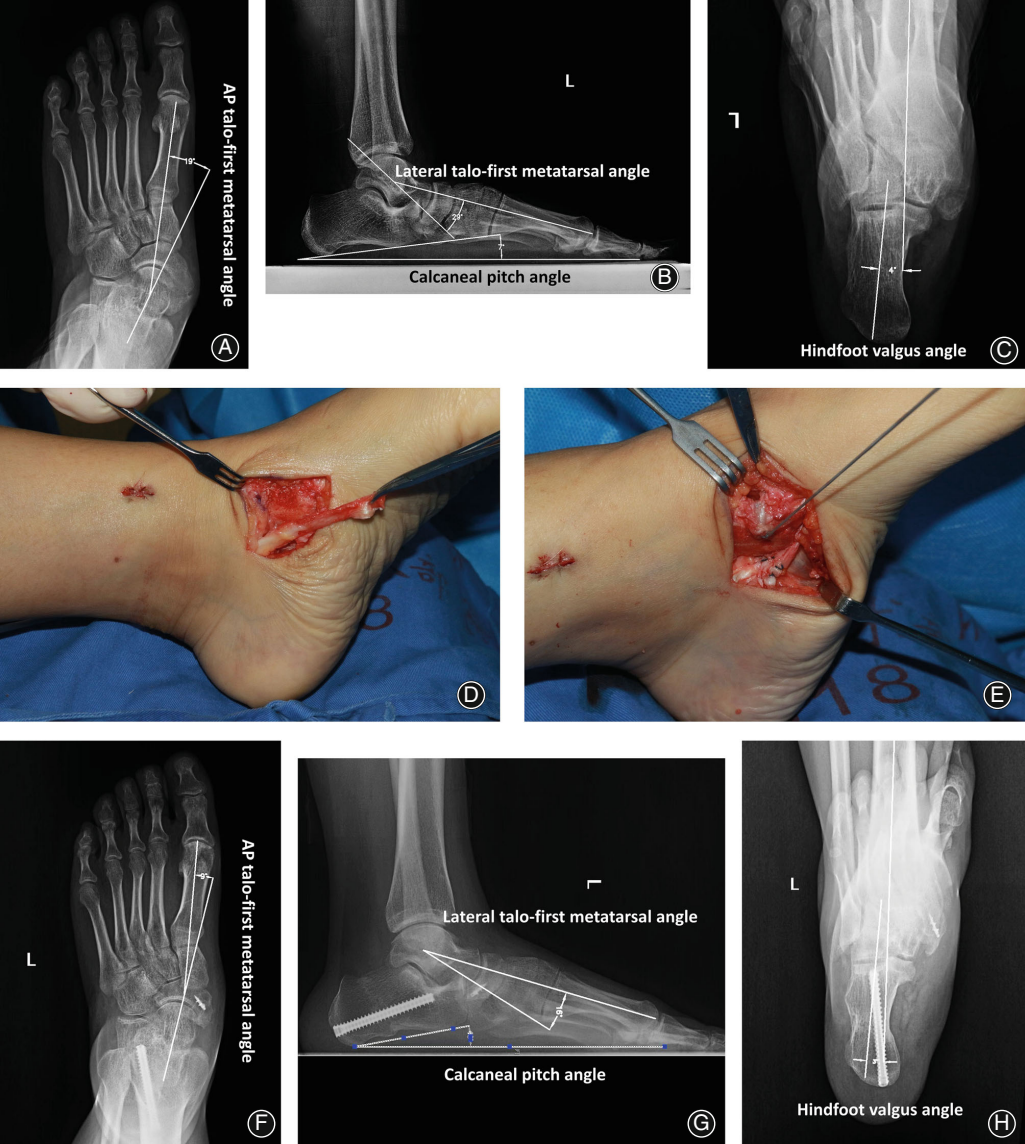
Correction of flexible flatfoot associated with symptomatic accessory navicular in a 27‐year‐old woman. Preoperative weight‐bearing radiographs illustrate the anteroposterior talo‐first metatarsal angle (A), lateral talo‐first metatarsal angle, calcaneal pitch angle (B), and hindfoot valgus angle (C). After treatment with calcaneal medial displacement osteotomy and the modified PTT reconstruction (D and E), the anteroposterior talo‐first metatarsal angle (F), lateral talo‐first metatarsal angle, calcaneal pitch angle (G), and hindfoot valgus angle (H) were improved significantly on postoperative weight‐bearing radiographs

**Fig. 3:**
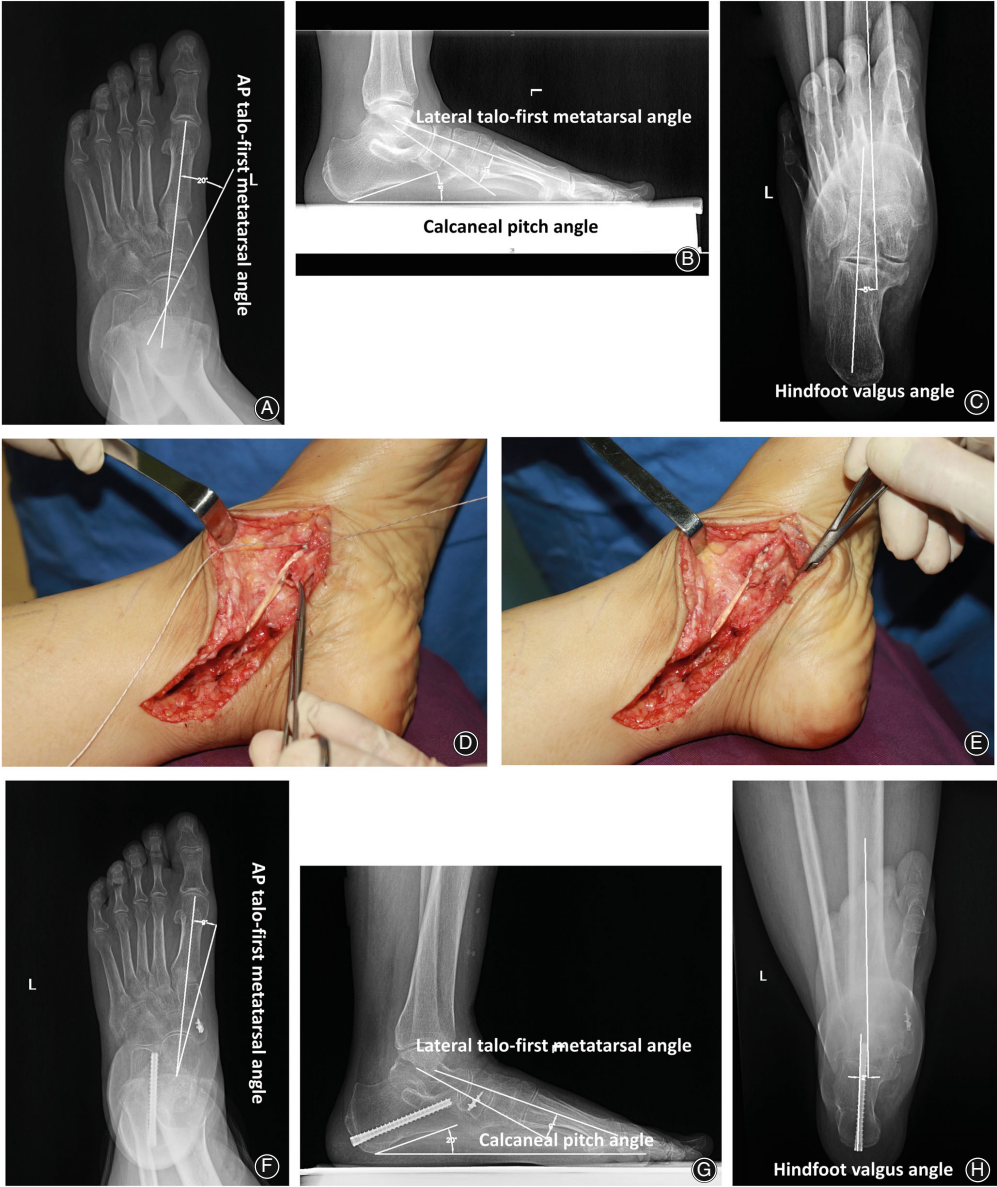
Correction of type IIA AAFD in a 55‐year‐old woman. Preoperative weight‐bearing radiographs represent anteroposterior talo‐first metatarsal angle (A), lateral talo‐first metatarsal angle, calcaneal pitch angle (B), and hindfoot valgus angle (C). After treatment with calcaneal medial displacement osteotomy and the modified PTT reconstruction (D and E), the anteroposterior talo‐first metatarsal angle (F), lateral talo‐first metatarsal angle, calcaneal pitch angle (G), and hindfoot valgus angle (H) were improved significantly on postoperative weight‐bearing radiographs

**Fig. 4:**
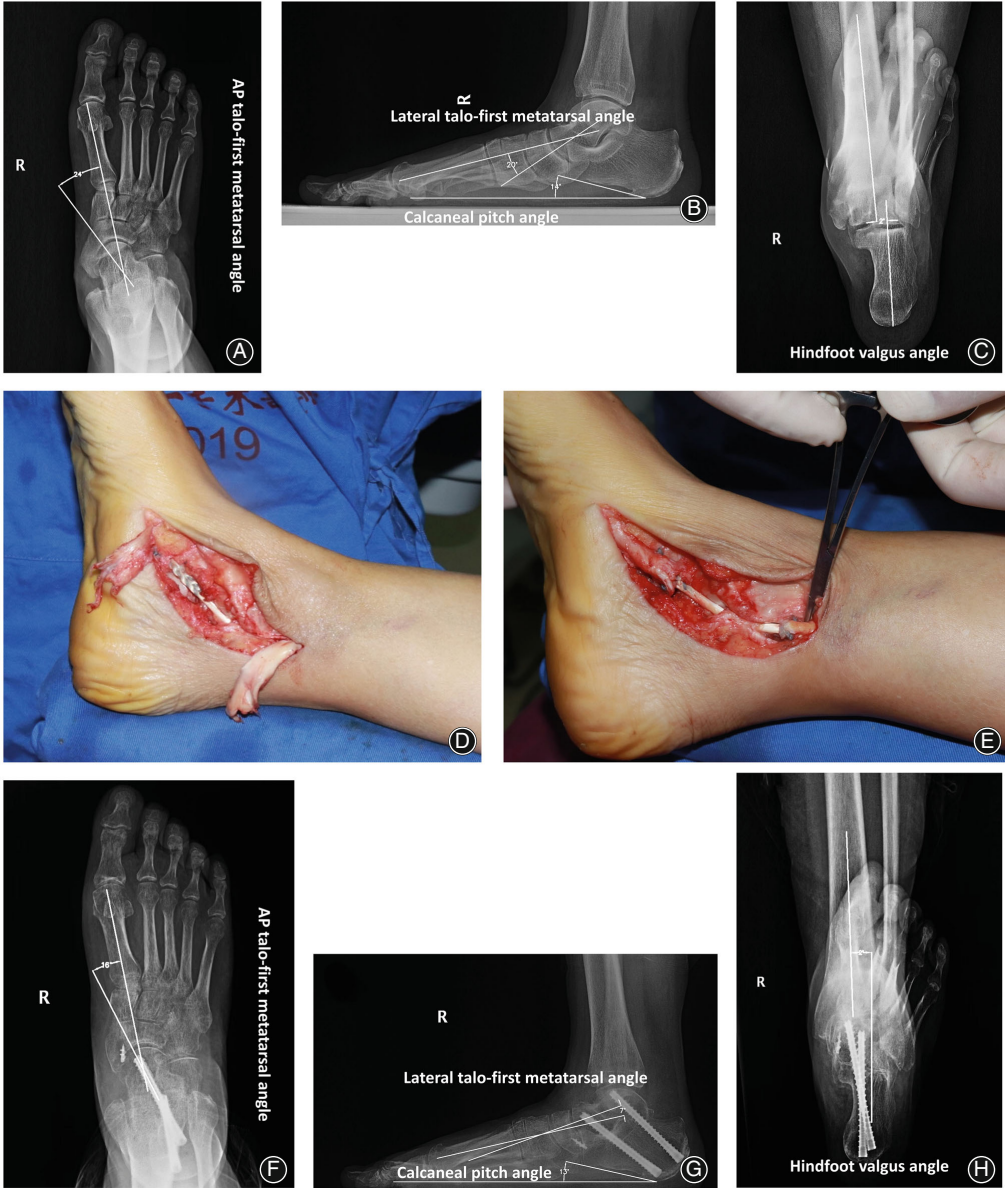
Correction of type IIA AAFD in a 56‐year‐old woman. Preoperative weight‐bearing radiographs demonstrate anteroposterior talo‐first metatarsal angle (A), lateral talo‐first metatarsal angle, calcaneal pitch angle (B), and hindfoot valgus angle (C). After treatment with calcaneal medial displacement osteotomy and the modified PTT reconstruction (D and E), the anteroposterior talo‐first metatarsal angle (F), lateral talo‐first metatarsal angle, calcaneal pitch angle, (G) and hindfoot valgus angle (H) were improved significantly on postoperative weight‐bearing radiographs

The current retrospective study comprised 16 patients with AAFD treated operatively. These 16 patients included three men and 13 women, and their mean age was 43.2 ± 15.1 years (age range: 21–64 years). In addition to the modified anatomic PTT reconstruction (Figure 1), all patients also underwent calcaneal medial displacement osteotomy (two typical cases are depicted in Figures 2 and 3) or subtalar arthrodesis (a typical case is illustrated in Figure 4).

### Surgical procedures

The standard modified PTT reconstruction (Figure 1) was performed following a bony procedure and gastrocnemius recession.

#### Anesthesia and Position

The patient was positioned supine for regional nerve block, and a thigh tourniquet was used.

#### Approach and Exposure

A medial incision was made immediately inferior to the medial malleolus and extended distally for 3–5 cm toward the navicular tuberosity, where the medial attachment was inserted in the tuberosity of the navicular, the inferior capsule of the naviculocuneiform joint, and the inferior surface of the medial cuneiform (Figure 1(A)). The PTT sheath was subsequently opened. After exploring the inferior surface of the medial cuneiform, where the PTT splits into its anterior and central branches, the splitting of the PTT was continued proximally for approximately 3 cm and then released (Figure 1(B)). Subsequently, the anterior branch was released off the navicular, and the diseased tendon was excised.

#### Posterior tibialis tendon reconstruction

For cases with enlarged tuberosities, partial tuberosity resection was first performed to normalize the size and shape of the tuberosity. Then, a classical FDL transfer was conducted in the navicular tuberosity (Figure 1(C)). Finally, the central band was sutured to FDL as tension was applied to tighten the plantar structures of the foot (Figure 1(D)).

### Postoperative treatment

The patients with bony procedures were allowed weight‐bearing after osteotomy healing at approximately 3 months postoperatively. The muscle‐strength training was initiated 3 months postoperatively.

### Evaluations Functional Evaluation

The patients underwent both preoperative and postoperative evaluations. As a clinical evaluation, a visual analogue scale (VAS) for pain and satisfaction and the American Orthopedic Foot and Ankle Society ankle–hindfoot scale (AOFAS‐AH) for function were applied. In pain VAS, a patient's pain ranges across a continuum from none (score 0) to an extreme amount of pain (score 10). In the satisfaction VAS, a patient's amount of satisfaction ranges across a continuum from no satisfaction (score 0) to extreme satisfaction (score 10). AOFAS‐AH is one of the most widely used assessment tools in foot surgery. This clinical rating system includes nine items divided into three sub‐scales: pain, function, and alignment. Each of the nine items was scored, thereby accumulating a total score ranging from zero points (indicating severe pain and impairment) to 100 points (no symptoms or impairment).

During the operation, the existence of the central bundle was detected and recorded, and the width of the central bundle was measured and recorded.

#### Isokinetic Strength Test

The plantarflexion and inversion strength were evaluated using the Biodex Isokinetic Dynamometer (Shirley, NY, USA), demonstrating a high test–retest reliability and superior performance to manual muscle testing.^15^, ^16^, ^17^ Peak torque ankle plantarflexion measurement was performed with the knee placed at 90° flexion, whereas the peak torque foot adduction measurement was performed with the ankle in 20° plantarflexion. Biodex testing was performed pre‐operatively, 1‐year postoperatively, and at a final follow‐up.

#### Radiographic Assessment

Preoperative and postoperative radiographic measurements included anteroposterior (AP) talo‐first metatarsal angle, percentage of talar head uncoverage, lateral talo‐first metatarsal angle, and calcaneal pitch angle.

#### Anteroposterior Talo‐first Metatarsal Angle

The AP talus‐first metatarsal angle was the angle between a line bisecting the anterior articular surface of the talus and a line bisecting the long axis of the first metatarsal bone on the AP foot‐weight‐bearing radiographs.

#### Percentage of Talar Head Uncoverage

The percentage of talar head uncoverage was the width of the uncovered talar head articular cartilage, as established by a line drawn proximal from the medial border of navicular cartilage divided by the total width of talar head articular cartilage.

#### Lateral Talo‐first Metatarsal Angle

The lateral talus‐first metatarsal angle was the angle between the line bisecting the long axis of the first metatarsal bone and a line drawn through the midpoints of the talar head and neck on the lateral foot‐weight‐bearing radiographs.

#### Calcaneal Pitch Angle

The calcaneal pitch angle was drawn between a line drawn along the edge of the plantar soft tissue shadow and a line drawn along the lower margin of the calcaneus on the lateral foot‐weight‐bearing radiographs.

### Statistical Analysis

Continual variables were expressed as mean ± standard deviation. The differences between the preoperative and follow‐up clinical features were compared by paired *t*‐test. Statistical significance was defined at *p* < 0.05. The statistical analysis was performed using the SPSS version 19 (IBM, Armonk, NY, USA).

## Results

### General Results

In addition to the modified anatomic PTT reconstruction, the patients underwent bone surgery, including medializing calcaneal osteotomy in 12 (75%) and subtalar joint fusion in four (25%). Ten patients underwent a gastrocnemius recession (62.5%). During surgery, the central branch was detected in all the cases, with an average width of 3.5 ± 0.8 mm (range, 3.1–4.3 mm).

### Function Outcomes

The average postoperative scores, including pain VAS, satisfaction VAS, total AOFAS‐AH, and all the sub‐scales of AOFAS‐AH, were considerably improved (Table 1).

**TABLE 1:** Functional outcome scores

Measurements	Preop	6 months*			Final follow‐up**		
	Scores	Scores	*t*	*p* value	Scores	*t*	*p* value
VAS pain	7.0 ± 1.0	3.1 ± 0.8	11.64	0.000	2.1 ± 1.6	9.067	0.000
VAS satisfaction	1.7 ± 1.1	6.5 ± 0.8	−13.774	0.000	8.0 ± 0.6	−17.96	0.000
AOFAS‐AH total score	39.8 ± 7.3	69.2 ± 6.7	−68.327	0.000	84.3 ± 4.8	−25.177	0.000
AOFAS‐AH pain	9.4 ± 8.3	24.4 ± 7.9	−5.975	0.000	32.5 ± 6.6	−9.459	0.000
AOFAS‐AH function	27.9 ± 5.9	35.3 ± 5.0	−7.515	0.000	43.0 ± 3.1	−12.209	0.000
AOFAS‐AH alignment	1.9 ± 2.4	8.8 ± 2.2	−11.000	0.000	8.8 ± 2.2	−11.000	0.000

Abbreviations: AOFAS‐AH, American Orthopedic Foot and Ankle Society ankle–hindfoot scale; VAS, Visual Analogue Scale.

*Significant Differences between scores pre‐operatively and 6‐month postoperatively (*p* < 0.001). **Significant Differences between scores pre‐operatively and final follow‐up (*p* < 0.001).

#### Pain Visual Analogue Scale

Improvement was observed in the pain VAS from the preoperative values of 7.0 ± 1.0 to the 6 months postoperative values of 3.1 ± 0.8 (*p* < 0.001), and lastly to final follow‐up values of 2.1 ± 1.6 (*p* < 0.001) (Table 1).

**TABLE 2:** Mean peak torque outcomes

	Angular velocity (°/s)	Mean peak torque (Nm)			
		Preop	Final follow‐up	*t*	*p*‐value
Plantarflexion					
	60	13.9 ± 4.5	12.9 ± 2.8	2.179	0.350
	120	12.8 ± 4.1	11.7 ± 3.5	1.698	0.098
Inversion					
	60	1.0 ± 0.4	3.9 ± 0.8	−2.970	0.008
	120	1.2 ± 0.2	3.6 ± 0.6	−1.378	0.007

#### Satisfaction Visual Analogue Scale

Satisfaction VAS improved from the preoperative values of 1.7 ± 1.1 to the 6‐month postoperative values of 6.5 ± 0.8 (*p* < 0.001 for all), and eventually to the values of 8.0 ± 0.6, (*p* < 0.001) at the final follow‐up (Table 1).

**TABLE 3:** Radiographic outcomes

Measurements	Preop	6 months*			Final follow‐up**		
	Scores	Scores	*t*	*p* value	Scores	*t*	*P* value
AP talo‐first metatarsal angle (°)	22.4 ± 3.2	14.1 ± 2.3	18.233	0.000	17.4 ± 4.6	9.663	0.000
Percentage of talar head uncoverage (%)	33.3 ± 10.7	17.8 ± 5.2	6.616	0.000	18.5 ± 4.0	5.902	0.000
Lateral talo‐first metatarsal angle (°)	22.7 ± 10.0	10.2 ± 4.6	5.751	0.000	10.7 ± 5.8	5.423	0.000
Calcaneal pitch angle (°)	8.9 ± 5.3	17.8 ± 6.3	−5.215	0.000	15.9 ± 7.1	−4.384	0.000

Abbreviations: AP, Anteroposterior.

*Significant Differences between scores pre‐operatively and 6‐month postoperatively (*p* < 0.001). **Significant Differences between pre‐operatively and final follow‐up (*p* < 0.001).

#### American Orthopaedic Foot and Ankle Society Ankle–Hindfoot Scale Score

The AOFAS‐AH total scale and the pain, function, and alignment sub‐scales of AOFAS‐AH, improved from the preoperative values of 39.8 ± 7.3, 9.4 ± 8.3, 27.9 ± 5.9, and 1.9 ± 2.4, respectively, to 6‐month postoperative values of 69.2 ± 6.7, 24.4 ± 7.9, 35.3 ± 5.0, and 8.8 ± 2.2 (*p* < 0.001 for all), and finally to final follow‐up values of 84.3 ± 4.8, 32.5 ± 6.6, 43.0 ± 3.1, and 8.8 ± 2.2 (*p* < 0.001 for all except alignment) (Table 1).

### Isokinetic Strength Testing Results

Since Biodex testing must be conducted in the hospital, all patients completed the Biodex study pre‐operatively, and 14 patients followed up for a Biodex study during the final follow‐up.

The operative extremity's preoperative average plantarflexion mean peak torque with the knee flexed at 90° was 13.9 ± 4.5 Nm at 60°/s angular velocity and 12.8 ± 4.1 Nm at 120°/s angular velocity. The mean peak torque exhibited no significant change more than one‐year post‐surgery, with values of 12.9 ± 2.8 Nm at 60°/s angular velocity (*p* = 0.350) and 11.7 ± 3.5 Nm at 120°/s angular velocity (*p* = 0.098). The operative extremity's preoperative average inversion mean peak torque with the ankle plantarflexed at 20° was determined to be 1.0 ± 0.4 Nm at 60°/s angular velocity and 1.2 ± 0.2 Nm at 120°/s angular velocity. The mean peak torque rose substantially more than 1‐year post‐surgery to 3.9 ± 0.8 Nm at 60°/s angular velocity (*p* = 0.008) and 3.6 ± 0.6 Nm at 120°/s angular velocity (*p* = 0.007) (Table 2).

### Radiographic Measurements

#### Anteroposterior Talo‐first Metatarsal Angle

The radiographic measurements of preoperative and postoperative films at more than 1‐year follow‐up revealed an improved AP talo‐first metatarsal angle, which decreased from 22.4 ± 3.2 degrees to 14.1 ± 2.3 degrees at 6‐months post‐surgery (*p* < 0.001) and 17.4 ± 4.6 degrees at the final follow‐up (*p* < 0.001)(Table 3).

#### Percentage of Talar Head Uncoverage

At 6‐months post‐surgery, the talar head uncoverage improved from 33.3% ± 10.7% to 17.8% ± 5.2% (*p* < 0.001) and 18.5% ± 4.0% at the final follow‐up (*p* < 0.001) (Table 3).

#### Lateral Talo‐first Metatarsal Angle

A reduction in the lateral talo‐first metatarsal angle, from 22.7 ± 10.0 degrees to 10.2 ± 4.6 degrees at 6‐months post‐surgery and 10.7 ± 5.8 degrees at the final follow‐up (all *p* < 0.001) (Table 3).

#### Calcaneal Ppitch Angle

In the lateral view, the calcaneal pitch angles altered from 8.9 ± 5.3 degrees to 17.8 ± 6.3 degrees at 6‐months post‐surgery (*p* < 0.001) and 15.9 ± 7.1 degrees at the final follow‐up (*p* < 0.001) (Table 3).

The postoperative radiographic outcomes at 6‐months post‐surgery persisted till the final follow‐up (>1 year).

### Complications

No wound complications or infections were observed. One patient developed a deep vein thrombosis 1 week after surgery and was finally cured after 2 weeks of using low‐molecular‐weight heparin calcium. At the most recent follow‐up, no patients have suffered a recurrence of flatfoot deformity, either clinically or radiographically.

## Discussion

### The Association between AAFD and PTT Dysfunction

PTT originates from the posterior tibia, fibula, and interosseous membrane. It runs posterior to the medial malleolus, arches plantar‐ward, and finally inserts into the navicular tuberosity, with multiple insertions scattered throughout the midfoot sole.^13^, ^14^ This anatomical characteristic of PTT is crucial for effective muscle power transmission.

PTT dysfunction has a close association with the adult‐acquired flatfoot deformity,^4^, ^5^, ^6^, ^7^ so PTT reconstruction is an essential procedure in the adult flexible flatfoot correction surgery.^10^, ^11^

### The Modified PTT Reconstruction in Restoring Plantar Insertions

Traditionally, extensive efforts have been made to achieve maximum PTT function although it is not so convenient to attain maximal limb function through PTT reconstruction.^18^, ^19^, ^20^, ^21^ Essentially, all the traditional techniques focus solely on the medial component reconstruction, ignoring the reconstruction of the other bands. PTT exhibits high morphological variability, and its insertions are primarily composed of three bundles mainly. Sarrafian and his colleagues described the anterior, central, and posterior components.^22^

In the study, the central branch was detected during the surgery in all cases in the current investigation, with an average width of 3.5 ± 0.8 mm (3.1–4.3 mm). Lewis discovered the importance of the central attachment to the medial origin of the flexor hallucis brevis and stated that it was a “uniquely human modification associated with the evolution of an arched, weight‐bearing foot”.^23^ In addition, the author further stated that it “clearly provides a mechanism for enhancing the contraction of the short hallucial flexor, precisely where the pull of an arch‐raising muscle is the most required.” These anatomical findings support the central band's critical role in maintaining the foot's longitudinal arch.

Because of the importance of the PTT central band in maintaining the foot longitudinal arch, the modified technique was developed in the study to restore the central band. As part of the method, a classical FDL transfer was performed in the navicular tuberosity. In certain cases with enlarged tuberosities, partial tuberosity resection was firstly performed to normalize the tuberosity's size and shape. The central band was then sutured to FDL when tension was applied to tighten the foot's plantar structures. The technique restored the plantar insertions.

### The Efficiency of the Modified PTT Reconstruction

Highlighting the success of the modified PTT reconstruction in the context of numerous other concomitant procedures is challenging. We endeavored to analyze its function and clinical efficacy to the best of our ability. After 6 months, the VAS pain and satisfaction scores improved significantly, albeit these scores did not reach the maximal outcome levels until 1 year. In addition, the AOFAS‐AH total score and AOFAS‐AH pain and function scores improved steadily from the pre‐operation stage through 6‐months post‐surgery to the final follow‐up. As a result, it would require at least 1 year to reap the maximum benefits of the procedure.

The Isokinetic dynamometry evaluation conducted in the present study did not demonstrate any statistically significant loss of the plantarflexion mean peak torque when a gastrocnemius recession was combined with the PTT reconstruction with FDL transfer final follow‐up. However, a significant progressive improvement in the inverted mean peak torque was observed at the final follow‐up (>1 year) in comparison to the ipsilateral extremity's preoperative mean peak torque. Finally, all radiographic measurements were observed to have improved significantly. As a result, it was concluded that the procedures, including the modified PTT reconstruction, could provide satisfactory outcomes.

### Limitations of the Study

According to anatomical research, the lateral bundle has a significant role in maintaining the medial longitudinal arch of the foot, albeit the linked conclusions have not been substantiated by biomechanical assessment. Previously, biomechanical studies focused on the association between the total tibialis posterior muscle and the AAFD. In terms of future research, a biomechanical study evaluating the change in the medial longitudinal arch height under cyclic axial loading with and without an activated lateral bundle of the tibial posterior tendon force has been proposed. Moreover, since it is difficult to demonstrate the efficacy of the modified PTT reconstruction in the context of numerous other concomitant procedures, it is impossible to infer that the unique approach utilized in the current study is superior to the old ones.

### Conclusions

It was sought to achieve a near‐anatomical location and function of PTT using the innovative modified approach utilized in this work, based on an awareness of its anatomical attachments. As a result, after more than a year of follow‐up, this technique consistently increases ankle inversion strength, medial longitudinal arch restoration, and satisfactory clinical outcomes. The biomechanical assessment of this reconstruction has been proposed and is being investigated.

## AUTHORS CONTRIBUTIONS

Ning Zhang, Yong Hu, and Dongsheng Zhou conceived of the presented idea. Yong Hu, Zhengxun Li and Zheng Huang performed the surgery. Ning Zhang, Yifan Wang and Wenpeng Xu contributed to the investigation of the outcomes post‐operatively. All authors discussed the results and contributed to the final manuscript.

## Funding

This research was supported by Key Research and Development Project of Shandong Province (No. 2019GSF108092), and Natural Science Foundation of Shandong Province (No. ZR2020MH090).

## References

[1] Daly PJ , Kitaoka HB , Chao EY . Plantar fasciotomy for intractable plantar fasciitis: clinical results and biomechanical evaluation. Foot Ankle. 1992;13:188–95. 1634150 10.1177/107110079201300404

[2] Palmanovich E , Shabat S , Brin YS , Feldman V , Kish B , Nyska M . Anatomic reconstruction technique for a plantar calcaneonavicular (spring) ligament tear. J Foot Ankle Surg. 2015;54:1124–6. 26253476 10.1053/j.jfas.2015.06.013

[3] Huang CK , Kitaoka HB , An KN , Chao EY . Biomechanical evaluation of longitudinal arch stability. Foot Ankle. 1993;14:353–7. 8406252 10.1177/107110079301400609

[4] Kitaoka HB , Luo ZP , An KN . Effect of the posterior tibial tendon on the arch of the foot during simulated weightbearing: biomechanical analysis. Foot Ankle Int. 1997;18:43–6. 9013114 10.1177/107110079701800109

[5] Kamiya T , Uchiyama E , Watanabe K , Suzuki D , Fujimiya M , Yamashita T . Dynamic effect of the tibialis posterior muscle on the arch of the foot during cyclic axial loading. Clin Biomech. 2012;27:962–6. 10.1016/j.clinbiomech.2012.06.006 22749639

[6] Pomeroy GC , Pike RH , Beals TC , Manoli A 2nd . Acquired flatfoot in adults due to dysfunction of the posterior tibial tendon. J Bone Joint Surg Am. 1999;81:1173–82. 10466651 10.2106/00004623-199908000-00014

[7] Kupcha PC , Shah SA . Posterior tibial tendon dysfunction as a cause of acquired flatfoot in adults. Del Med J. 1997;69:255–7. 9170703

[8] Ling SK , Lui TH . Posterior tibial tendon dysfunction: An overview. Open Orthop J. 2017;11:714–23. 28979585 10.2174/1874325001711010714 PMC5620404

[9] Coetzee JC , Hansen ST . Surgical management of severe deformity resulting from posterior tibial tendon dysfunction. Foot Ankle Int. 2001;22:944–9. 11783917 10.1177/107110070102201202

[10] Wukich DK , Rhim B , Lowery NJ , Dial D . Biotenodesis screw for fixation of FDL transfer in the treatment of adult acquired flatfoot deformity. Foot Ankle Int. 2008;29:730–4. 18785425 10.3113/FAI.2008.0730

[11] Charwat‐Pessler CG , Hofstaetter SG , Jakubek DE , Trieb K . Interference screw for fixation of FDL transfer in the treatment of adult acquired flat foot deformity stage II. Arch Orthop Trauma Surg. 2015;135:1369–78. 26204981 10.1007/s00402-015-2295-6

[12] Hui HE , Beals TC , Brown NA . Influence of tendon transfer site on moment arms of the flexor digitorum longus muscle. Foot Ankle Int. 2007;28:441–7. 17475138 10.3113/FAI.2007.0441

[13] Bloome DM , Marymont JV , Varner KE . Variations on the insertion of the posterior tibialis tendon: a cadaveric study. Foot Ankle Int. 2003;24:780–3. 14587993 10.1177/107110070302401008

[14] Olewnik L . A proposal for a new classification for the tendon of insertion of tibialis posterior. Clin Anat. 2019;32:557–65. 30758860 10.1002/ca.23350

[15] Aydog E , Aydog ST , Cakci A , Doral MN . Reliability of isokinetic ankle inversion‐ and eversion‐strength measurement in neutral foot position, using the Biodex dynamometer. Knee Surg Sports Traumatol Arthrosc. 2004;12:478–81. 15156307 10.1007/s00167-004-0530-8

[16] Kou JX , Balasubramaniam M , Kippe M , Fortin PT . Functional results of posterior tibial tendon reconstruction, calcaneal osteotomy, and gastrocnemius recession. Foot Ankle Int. 2012;33:602–11. 22835399 10.3113/FAI.2012.0602

[17] Tankevicius G , Lankaite D , Krisciunas A . Test‐retest reliability of biodex system 4 pro for isometric ankle‐eversion and ‐inversion measurement. J Sport Rehabil. 2013;22:212–5. 23579478 10.1123/jsr.22.3.212

[18] Senses I , Kiter E , Gunal I . Restoring the continuity of the tibialis posterior tendon in the treatment of symptomatic accessory navicular with flat feet. J Orthop Sci. 2004;9:408–9. 15278781 10.1007/s00776-004-0793-4

[19] Backus JD , McCormick JJ . Tendon transfers in the treatment of the adult flatfoot. Foot Ankle Clin. 2014;19:29–48. 24548507 10.1016/j.fcl.2013.11.002

[20] Vaudreuil NJ , Ledoux WR , Roush GC , Whittaker EC , Sangeorzan BJ . Comparison of transfer sites for flexor digitorum longus in a cadaveric adult acquired flatfoot model. J Orthop Res. 2014;32:102–9. 24115238 10.1002/jor.22488

[21] Mann RA . Posterior tibial tendon dysfunction. Treatment by flexor digitorum longus transfer. Foot Ankle Clin. 2001;6:77–87. 11385929 10.1016/s1083-7515(03)00080-9

[22] Sarrafian SK . Anatomy of the Foot and Ankle. 2nd ed. Philadelphia: J.B. Lippincott; 1993. p. 204–5.

[23] Lewis OJ . The Tibialis posterior tendon in the primate foot. J Anat. 1964;98:209–18. 14154423 PMC1261276

